# Increased anxiety and stress-related visits to the Shanghai psychiatric emergency department during the COVID-19 pandemic in 2020 compared to 2018–2019

**DOI:** 10.3389/fpsyt.2023.1146277

**Published:** 2023-03-23

**Authors:** TianHong Zhang, Zheng Chen, XuDong Xiao, LinLin Zhou, YeGang Hu, LiHua Xu, YanYan Wei, XiaoChen Tang, HaiChun Liu, Tao Chen, HaiSu Wu, XuMing Wu, JiJun Wang

**Affiliations:** ^1^Shanghai Intelligent Psychological Evaluation and Intervention Engineering Technology Research Center (20DZ2253800), Shanghai Key Laboratory of Psychotic Disorders, Shanghai Mental Health Center, Shanghai Jiaotong University School of Medicine, Shanghai, China; ^2^Department of Automation, Shanghai Jiao Tong University, Shanghai, China; ^3^Big Data Research Lab, University of Waterloo, Waterloo, ON, Canada; ^4^Labor and Worklife Program, Harvard University, Cambridge, MA, United States; ^5^Nantong Fourth People's Hospital and Nantong Brain Hospital, NanTong, Jiangsu, China; ^6^Center for Excellence in Brain Science and Intelligence Technology (CEBSIT), Chinese Academy of Science, Shanghai, China; ^7^Brain Science and Technology Research Center, Shanghai Jiao Tong University, Shanghai, China

**Keywords:** psychiatric hospital, stress, anxiety disorder, pandemic, emergency psychiatry

## Abstract

**Background:**

The coronavirus disease 2019 (COVID-19) pandemic has had a significant and far-reaching impact on mental health. The psychiatric emergency department (PED) is pivotal in the management of acute and severe mental illnesses, especially anxiety-and stress-related disorders.

**Aims:**

This study aimed to evaluate whether changes in the frequency or patients’ demographics of visiting the PED occurred during the COVID-19 pandemic among individuals with anxiety and stress-related disorders.

**Methods:**

This cross-sectional study used data on PED visit counts from the largest psychiatric hospital in China between 2018 and 2020 (before and during the COVID-19 pandemic). Data from 2020, representing the COVID-19 pandemic period, were extracted from electronic medical records and compared using descriptive statistics for the same periods in 2018 and 2019.

**Results:**

The number of PED visits related to anxiety and stress disorders per year increased from 83 in 2018 to 136 (63.9% increase) in 2019 and 239 (188.0% increase) in 2020. Compared to that in 2018 and 2019, the proportion of PED visits in 2020 among patients with anxiety and stress disorders increased significantly. Patients with anxiety-and stress-related disorders during PED visits in 2020 were younger than those in 2018 and 2019 (three-year groups: *F* = 9.124, *df* = 2, *p* < 0.001).

**Conclusion:**

Despite the epidemic-policy barriers against PED visits, PED care seeking has increased, thereby underscoring the need for crisis prevention services for patients with stress and anxiety disorders.

## Introduction

The coronavirus disease 2019 (COVID-19) pandemic has caused anxiety and stress-related psychosocial disruption in response to the threat of disease (1). The unpredictable emergence of COVID-19 cases in China and the impact of the pandemic on psychological health may persist for a long time (2). The impact of not only its rapid spread but also the changes in people’s daily lives characterized as guarded and distant (3), the devastating impact on the economy, and the profound impact on people’s sense of security and uncertainty about the future has been significant (4).

Evidence indicates that the COVID-19 pandemic has generated substantial increases in the incidence of depression, anxiety, and acute stress disorders (5–7). There is widespread consensus that mental health problems are increasing and are mainly caused by the COVID-19 pandemic, particularly due to social isolation, fear of infection, parental distress, and family financial stress (8, 9). The COVID-19 pandemic has been recognized as an important threat to mental health and well-being (10, 11). However, these findings were largely derived from self-reporting surveys, and only few studies have documented real cases with several severe mental disorders, such as suicidal and violent behavior before and during the pandemic.

Many investigations have been designed to survey the general population to assess the degree of psychological impact of the pandemic (12–14); however, this does not directly support the increase in mental illness. Psychiatric emergency departments (PED) are at the forefront of treating the mental illness crisis, and changes in PED visits during the COVID-19 pandemic provide more direct evidence to evaluate the psychological impact of the pandemic. Changes in PED visits have been used to understand the potential impact of COVID-19 on serious mental disorder outcomes (15, 16). Unfortunately, the research results are not consistent in different regions owing to the different stages of the epidemic and control policies. The number of PED visits could be decreased by COVID-19 stay-at-home orders (17) or fear of COVID-19 infection (18, 19), varied by regions (20), and increased by the deconfinement period (21).

## Methods

### Participants

This cross-sectional study included all patients with anxiety-and stress-related mental illnesses who visited the Shanghai Mental Health Center (SMHC) PED between 2018 and 2020 (before and during the COVID-19 pandemic). The emergency department at the SMHC is the largest PED and provides 24-h service all year-round. It is the only referral hospital in Shanghai to serve all emergency psychiatric patients. The PED visits for F4 (neurotic, stress-related, and somatoform disorders, F40 - F48) diagnoses according to the 10^th^ revision of the International Classification of Diseases and Related Health Problems (ICD-10) were extracted from the SMHC Diagnosis and Treatment System Database. The SMHC Research Ethics Committee approved the data analysis. This study should be considered a public health surveillance rather than a research study involving human subjects; therefore, informed consent was waived for these secondary data analyses. All data were anonymized before analysis.

### Setting

The PED at SMHC is the largest mental health clinic offering medication management and psychological crisis interventions in Shanghai and China. PED patients are mainly from Shanghai and from different parts of the country. In total, the PED at SMHC reported 1,767, 2,210, and 2,648 visits in 2018, 2019, and 2020, respectively. Approximately 1,000 professional staff members provide care to patients at the SMHC. Among them, 258 were psychiatrists and psychologists and 541 were psychiatric nurses, along with other support staff members. PED provides comprehensive clinical services, including psychological assessments and counselling, medical management, brain stimulation, crisis intervention, and hospitalization. Patients seek help for issues ranging from general mental illnesses (such as schizophrenia and bipolar disorder) to more severe crises (such as suicide attempts and medication overdose). The PED is composed of 13 senior psychiatrists and 15 experienced nurses. The psychiatrists must have the professional title of attending doctor or above, and the nurses must have more than 5 years of work experience.

### COVID-19 pandemic and related policy in Shanghai

**Figure 1:**
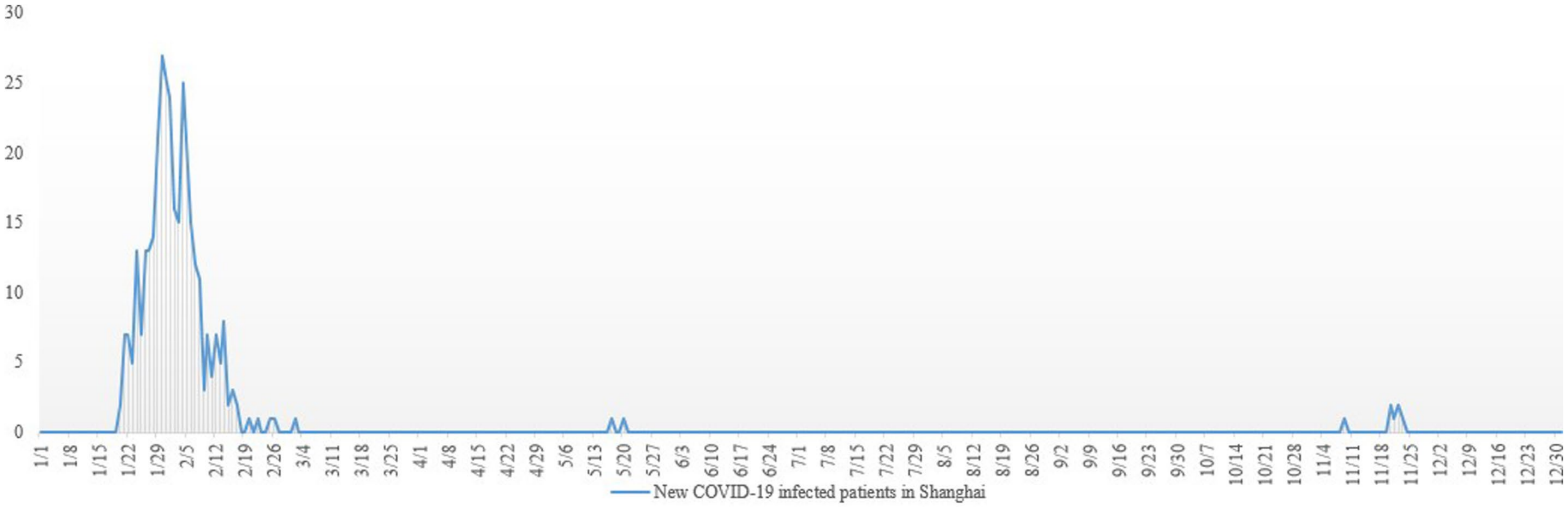
The number of COVID-19 infected people in Shanghai from January 1, 2020, to December 31, 2020.

Shanghai was a typical city during the epidemic. First, Shanghai had a relatively small number of local COVID-19 cases and a high recovery rate in 2020, making it a role model for other major cities and provinces in China (21). The epidemic data were obtained from the official data released by the National Health Commission of China and the Shanghai Municipal Health Commission for the number of COVID-19 infected people from 0:00 on January 1, 2020, to 24:00 on December 31, 2020. A total of 348 new COVID-19 cases were reported in 2020. The detailed distribution of COVID-19 cases in 2020 is presented in Figure 1. Second, Shanghai is one of China’s largest, most populated (24,882,000 in 2020), and most internationalized cities. Shanghai’s psychiatric emergency strategies and experiences can be useful in other large cities worldwide. Third and most importantly, Shanghai has the best medical resources in China, and many patients come for medical treatment from other provinces. The Shanghai government has always adopted a strategy of accurate epidemic prevention during the epidemic period, with remarkable epidemic prevention effects and a stable medical environment.

### Diagnostic categories

The SMHC electronic diagnosis and treatment system was designed according to the ICD-10. In the current analysis, the F4 (F40 – F48) neurotic, stress-related, and somatoform disorders were selected. Three diagnostic categories were classified as follows: anxiety disorders, stress-related disorders, and somatoform and dissociative disorders. The category of anxiety disorders included F40 phobic anxiety disorders and F41 other anxiety disorders (such as panic disorder and generalized anxiety disorder). The category of stress-related disorders included F43 acute stress reactions, post-traumatic stress disorder, and adjustment disorders. The category of somatoform and dissociative disorder included F44 dissociative (conversion) and somatoform disorders.

### Statistical methods

The PED visit counts are presented and compared according to age, sex, and diagnostic categories. Descriptive statistical analyses, independent sample t-tests, chi-square tests, and one-way analysis of variance (ANOVA) were performed to assess temporal trends between 2018, 2019 and 2020, using IBM SPSS Statistics v.16 for Windows (IBM Corp., Armonk, NY, IBM Corp). All datasets were transferred to Excel spreadsheets (Microsoft Corporation, Redmond, WA, USA), and pie and bar graphs were generated using the software. Statistical significance was set at *p* < 0.05.

## Results

**Figure 2:**
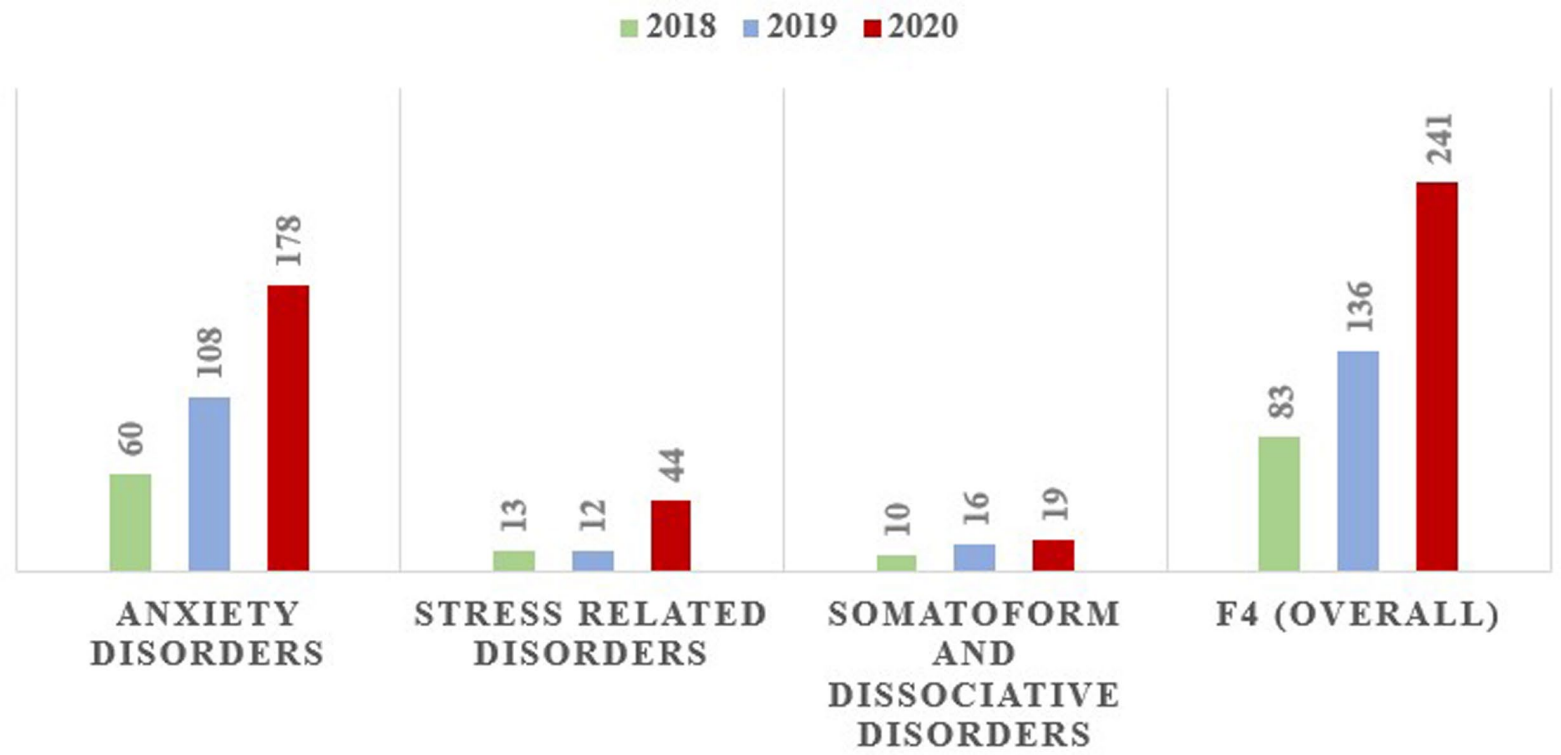
The number of yearly F4-PED visits across 2018, 2019, and 2020.

From January 1, 2018, to December 31, 2020, a total of 458 PED visits for F4 diagnoses were recorded in the SMHC administrative dataset. Among them, 296 individuals (64.6%) were female and 162 (35.4%) were male, with a mean age of 43.4 years (SD = 16.5), (range, 11–86 years). The number of F4-PED visits per year increased from 83 in 2018 to 136 (63.9% increase) in 2019 and 239 (188.0% increase) in 2020 (Figure 2).

To further compare changes in the distribution of F4 diagnostic categories between 2020 and 2018–2019, Figure 3 shows the annual proportion of patients who visited the PED in each diagnostic category. The proportions of the three diagnostic categories were not significantly different among the 3 years (*χ^2^* = 7.597, *df* = 4, *p* = 0.108).

**Figure 3:**
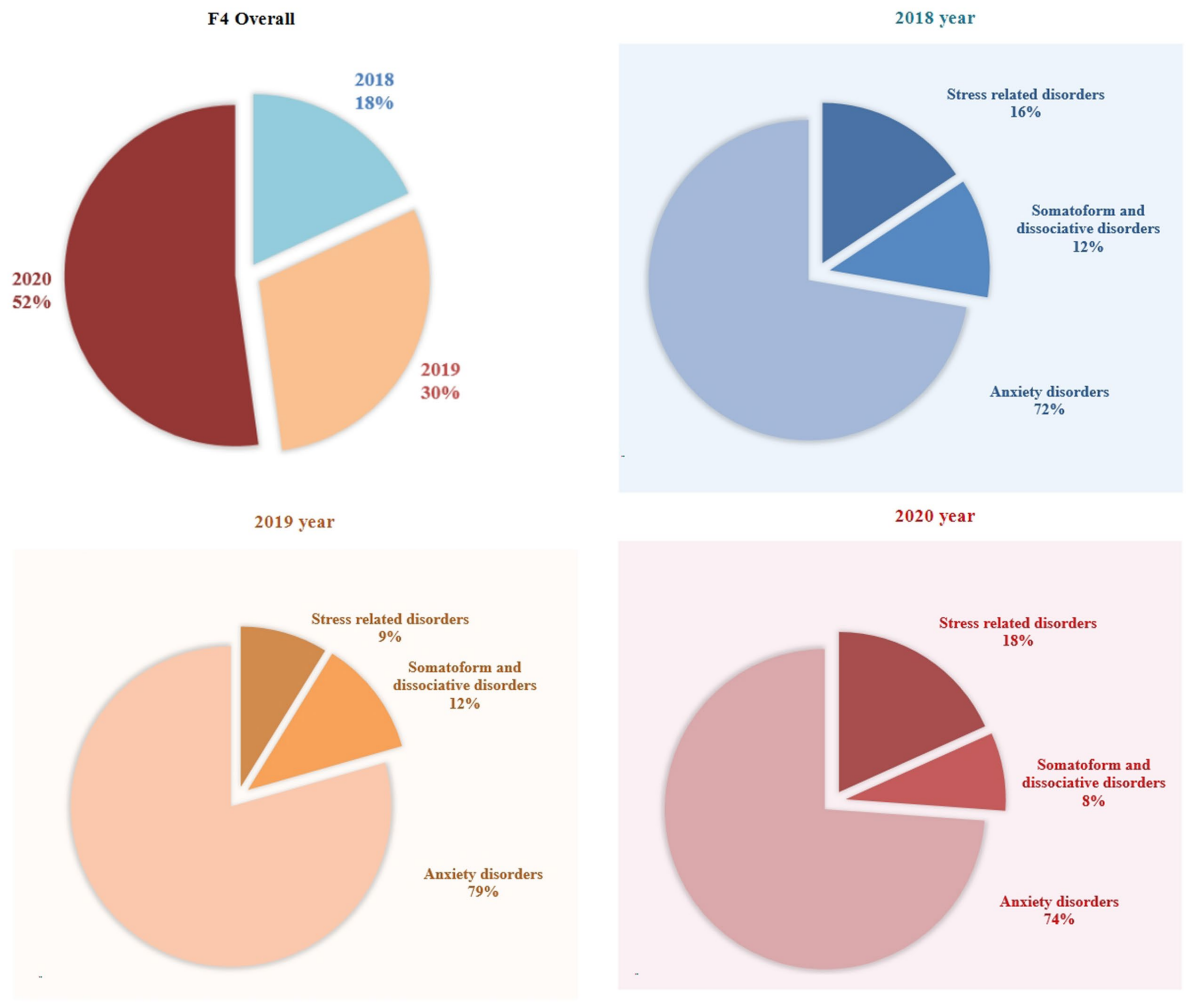
The proportion of F4 diagnostic categories in yearly PED visits across 2018, 2019, and 2020.

**Figure 4:**
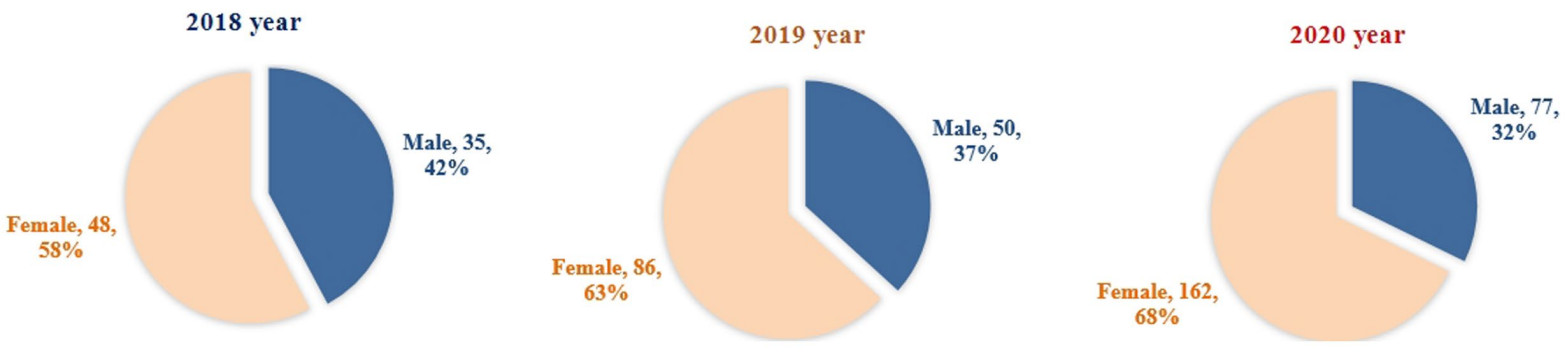
The proportion of sex in yearly F4-PED visits across 2018, 2019, and 2020.

**Figure 5:**
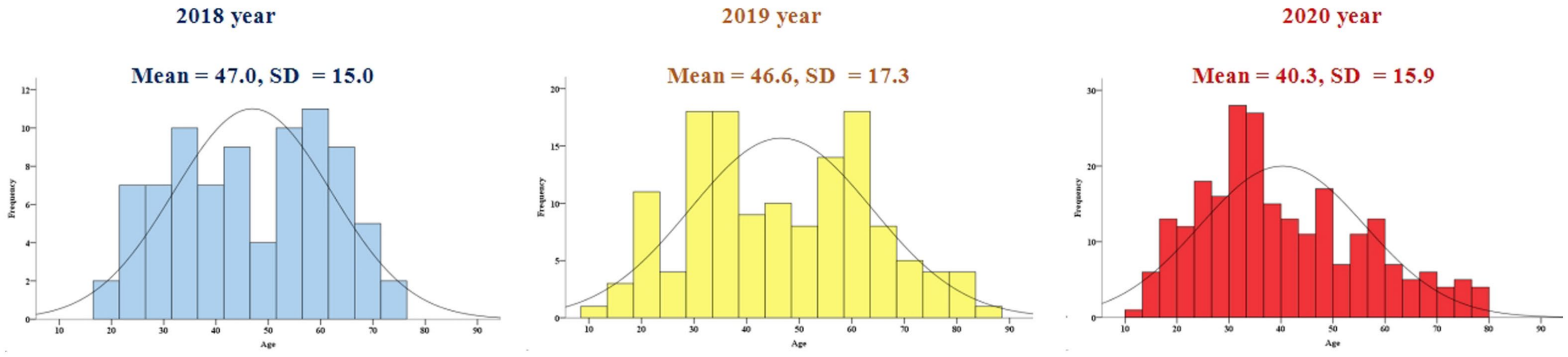
The distribution of age in yearly F4-PED visits across 2018, 2019, and 2020.

The sex proportion and age distribution across the 3 years are presented in Figures 4, 5. Although the proportion of women is increasing, the sex proportion in F4-PED visits from 2018 to 2020 was not statistically significant (*χ*^2^ = 2.833, *df* = 2, *p* = 0.243).

Patients in the 2020 F4-PED visits were younger than those in 2018 and 2019 (three-year groups: *F* = 9.124, *df* = 2, *p* < 0.001). Patients with F4 diagnosis were grouped into young age group and old age group based on the age of 30 and 18, and compare the proportion of the two groups in the 3 years from 2018 to 2020. When grouping by the age of 30, the proportion of young patients was 18.1% in 2018, 18.4% in 2019, but increased to 27.6% in 2020 (2018 vs. 2020, *χ*^2^ = 2.980, *df* = 1, *p* = 0.084; 2019 vs. 2020, *χ*^2^ = 4.020, *df* = 1, *p* = 0.045). When grouping by the age of 18, the proportion of young patients was 0% in 2018, 2.9% in 2019, but increased to 5.0% in 2020.

## Discussion

To the best of our knowledge, this cross-sectional study is the first to provide hospital encounter data, demonstrating a potential association between the pandemic and increased anxiety-and stress-related PED visits in Shanghai. Anxiety-and stress-related disorders exhibited greater increases in overall visits in 2020 than in 2018 or 2019. This growth makes the number of patients in 2020 greater than the sum of the previous 2 years, suggesting an increase in the burden of these disorders during the pandemic. These findings are comparable to the prevalence reported in studies conducted in other countries during the COVID-19 pandemic (22, 23), revealing that despite some variations, the overall clinical picture of pandemic-related anxiety and stress symptoms is universal.

Although affected by fear of contagion and epidemic control measures, PED visits for anxiety-and stress-related disorders in Shanghai were more frequent during the pandemic than during the homologous pre-pandemic period. This result is inconsistent with those of previous reports (17, 18, 20, 24), which found that the overall ED visits have decreased. These studies often need to explain the contradictions through assumptions; that is, the recognition of the epidemic leads to an increase in people’s psychological stress and pressure (1, 5), and social isolation leads to an increase in psychological problems (25, 26). Meanwhile, the number of PED visits is reduced owing to the fear of infection. However, in the current study, under the infection rate and prevention and control measures taken at the time of the epidemic in Shanghai, the number of PED visits increased consistently. These findings confirmed the increased mental health burden and help-seeking behavior under normalized epidemic management after a relatively serious epidemic in the early stages.

Moreover, in a secondary analysis of age, our findings suggested that younger people are a vulnerable population (27, 28) to anxiety and stress disorders during the pandemic. During quarantine, children and adolescents experienced periods without school, causing decreased physical activity, more internet time, irregular sleep patterns, and less appropriate diets, which have negative and potentially prolonged effects on the psychological health of individuals (29). Teenagers who have never experienced a pandemic in their lifetime may be less mature and perhaps incapable of facing this lifestyle transformation; for instance, students had to complete their school curriculum online. Evidence suggests that reciprocal interactions between brain functions and social activities and quarantine may have negative impacts on anxiety and stress-related disorder onsets during adolescence (30).

Given the potentially serious consequences of untreated anxiety and stress-related symptoms on psychological outcomes in adolescents, interventions are urgently needed to reduce symptoms and build resilience. Before these patients seek emergency assistance, psychological interventions to prevent and treat anxiety and stress-related disorders are effective, with cognitive behavior therapy (CBT) emerging as a front-line treatment that potentially offers additional benefits to reduce anxiety and increase social support (31, 32). To reduce the risk of spreading infections, many CBT practitioners have turned to digital therapies (33). CBT delivered *via* the Internet (iCBT) shows treatment effectiveness comparable to that of CBT (34, 35) and is cost-effective (36).

The present study has some limitations. First, only one site was included in this study; the data were not nationally representative, and the results may not be generalizable to populations in other areas. The degree of development of the city and epidemic scale are different. Second, given that standardized structured interviews cannot be conducted in the emergency environment, the diagnostic categories may be incomplete or inaccurate when the first visits are recorded. Third, PED visits should not be interpreted as equal to the overall mental health burden because many patients with mental disorders do not visit the PED. Fourth, some other factors, such as economic status and employment policy, which might confound the relationship between epidemic scale and PED visits, were not controlled in our study.

## Conclusion

In summary, these findings suggest that the COVID-19 pandemic is associated with an increase in anxiety and stress disorders, especially in younger groups, which may require public attention and support by implementing special interventions or prevention programs (37, 38).

## Data availability statement

The raw data supporting the conclusions of this article will be made available by the authors, without undue reservation.

## Ethics statement

Ethical approval for the study was obtained from the Human Research Ethics Committee at the Shanghai Mental Health Center (Ref No: 2021-14). This study should be considered a public health surveillance rather than a research study involving human subjects; therefore, informed consent was waived for these secondary data analyses. All data were anonymized before analysis.

## Author contributions

TZ, ZC, XX, HW, and JW conceptualized the study, wrote the first draft of the manuscript, and conducted statistical analyses. XX, LZ, LX, and YW collected and organized the primary data. YH, HL, TC, and XT managed the literature search, statistical analyses, and edited the manuscript. XW, JW, and TZ designed the study and supervised its implementation. All authors contributed to the article and approved the submitted version.

## Funding

This study was supported by the National Natural Science Foundation of China (82151314, 81901832, 81871050, 82171497, 82151314, and 82101623), Science and Technology Commission of Shanghai Municipality (21S31903100, 22Y11903800, 2018SHZDZX01, and 2018SHZDZX05) and ZJLab, Shanghai Clinical Research Center for Mental Health (19MC1911100), and Clinical Research Plan of SHDC (SHDC2022CRD026 and SHDC2020CR4066).

## Conflict of interest

The authors declare that the research was conducted in the absence of any commercial or financial relationships that could be construed as a potential conflict of interest.

## Publisher’s note

All claims expressed in this article are solely those of the authors and do not necessarily represent those of their affiliated organizations, or those of the publisher, the editors and the reviewers. Any product that may be evaluated in this article, or claim that may be made by its manufacturer, is not guaranteed or endorsed by the publisher.
